# Does Orally-Administered Radiocontrast Impair Ultrasound Image Quality in Pediatric Patients?

**DOI:** 10.5811/westjem.2019.10.44104

**Published:** 2020-02-21

**Authors:** Amit Patel, Marla Levine, Eitan Dickman, Lawrence Haines, Peter Homel, Antonios Likourezos, Illya Pushkar, Jefferson Drapkin, Alexander Arroyo

**Affiliations:** Maimonides Medical Center, Department of Emergency Medicine, Brooklyn, New York

## Abstract

**Introduction:**

It is commonly assumed that orally-administered radiocontrast material (ORC) preceding abdominal ultrasound (US) performance can obscure image quality and potentially impair diagnostic accuracy when assessing patients with abdominal pain. Due to this concern, ORC administration per protocol for computed tomography (CT) is often delayed until after US performance, potentially contributing to prolonged length of stay in the emergency department (ED) in patients with concern for abdominal pathology. The objective of this study was to evaluate whether early administration of ORC in children with abdominal pain receiving abdominal CT for possible appendicitis obscures subsequent abdominal US image quality.

**Methods:**

We designed a prospective observational study of children <18 years of age presenting to a pediatric ED with abdominal pain who were set to receive ORC prior to obtaining an abdominal CT. These patients received a point-of-care ultrasound (POCUS) of the abdomen to assess the abdominal aorta and right lower quadrant (RLQ) structures (psoas muscle and iliac vessels) pre- and post-ORC administration. Images were compared independently by two blinded emergency US-certified physician-assessors for quality, specifically to determine whether ORC obscured the anatomical structures in question.

**Results:**

A total of 17 subjects were enrolled, and each subject had two POCUS studies of the abdomen, one pre- and one post-ORC administration looking to visualize the anatomy of the RLQ and abdominal aorta in both studies. Statistical analysis showed no significant differences in mean values of POCUS image quality scoring by two blinded US-trained physician-assessors for either RLQ structures or abdominal aorta when performed pre- and post-administration of ORC.

**Conclusion:**

Early ORC administration in children with abdominal pain does not adversely affect image quality of a subsequently performed abdominal US. Patients who may require abdominal CT to determine the etiology of abdominal pain can receive early administration of ORC prior to US performance to help minimize ED length of stay without impairing US diagnostic accuracy.

## INTRODUCTION

Abdominal pain is a common pediatric outpatient complaint, accounting for 5–10% of all pediatric emergency department (ED) visits.^1^ The differential diagnosis can range from benign conditions to surgical emergencies, or to potentially catastrophic conditions such as malrotation with midgut volvulus. The ability to expeditiously evaluate and accurately diagnose a patient with appendicitis can be challenging and time intensive. Delaying time to diagnosis in appendicitis can lead to perforation, while expeditiously managing patients who require a stepwise approach to pediatric abdominal pain can minimize time to diagnosis and length of stay in a busy ED. Historically, oral and/or rectal contrast has been used when performing abdominal computed tomography (CT) due to the non-opacification in luminal obstruction, such as that that seen in an inflamed or obstructed appendix.

There is a theoretical concern that the presence of orally administered radiocontrast (ORC) in the gastrointestinal tract could obscure ultrasound (US) image quality and therefore potentially affect diagnostic accuracy in evaluating patients with abdominal pain. Thus, ORC administration is often delayed until after US performance. This delay can contribute to inefficient patient flow, prolonged ED length of stay, and ultimately an increase in time to diagnosis. We chose to use the psoas muscle, iliac vessels, and abdominal aorta as the landmarks in this study because these structures are readily and easily identifiable and would be presumed to be obscured by ingested oral contrast in the bowel that overlies these organs. If these structures were easily identified on the same patient in both pre- and post-ORC point-of-care ultrasound (POCUS) images, then we can conclude that administration of oral contrast does not affect US image quality and can be administered as early as possible. This can reduce wait time to CT and shorten time to diagnosis in patients with abdominal pain potentially leading to less complications. We know of no such study that has looked at this issue in children.

The purpose of this study was to determine whether ORC administration in children with abdominal pain affects the image quality of a subsequently performed abdominal US, either POCUS or formal radiological study.

## STUDY DESIGN AND SETTING

We conducted a prospective observational study on children <18 years old who presented to the pediatric ED (PED) between June 2014 and March 2016. We used a convenience sample, as eligible children were screened whenever study personnel were available. The study was approved by the hospital’s institutional review board. Children with abdominal pain were selected if there was a consideration for abdominal pathology requiring US and, if not diagnostic, subsequent CT. The hospital’s PED is located in Brooklyn, New York, with an annual patient volume of approximately 36,000; of that total, approximately 200 children are diagnosed with appendicitis each year.

**Population Health Research Capsule**

What do we already know about this issue?

It is thought that orally administered radiocontrast (ORC) before abdominal ultrasound (US) can obscure image quality and impair diagnostic accuracy in patients with abdominal pain.

What was the research question?

Does ORC in children receiving computed tomography (CT) for appendicitis obscure subsequent abdominal US images?

What was the major finding of the study?

Early ORC in children does not adversely affect image quality of a subsequently performed abdominal US.

How does this improve population health?

Patients requiring CT to determine the etiology of abdominal pain can receive ORC prior to US to help minimize ED length of stay without impairing US diagnostic accuracy.

Prior to initiating the study, all participating emergency physicians in the PED received formal instruction in performing POCUS examinations of the abdominal aorta and right left quadrant (RLQ) structures (psoas muscle and iliac vessels) or received in-service training on POCUS examination of the abdominal aorta and RLQ structures. Prior to enrolling patients, the non-fellowship trained physicians were also required to perform 25 scans in which image quality was evaluated and approved by physicians in the division of emergency US. There were three enrolling physicians: two were pediatric point-of-care emergency ultrasound fellowship-trained attendings and one was a pediatric emergency medicine (PEM) fellow with no prior background in emergency US. The PEM fellow researcher received in-service training on POCUS examination of the abdominal aorta and RLQ structures, and prior to enrolling patients was also required to perform 25 of these scans in which image quality was evaluated and approved by physicians in the division of emergency ultrasound. POCUS examinations were performed with a Siemens Zonare Z.one Ultra (Zonare Medical Systems, Inc. Mountain View, California) US machine, using a curvilinear probe (6–2 megahertz [MHz] transducer) and/or a linear probe (8–10 MHz transducer).

### Inclusion and Exclusion Criteria

Children met inclusion criteria if they were <18 years old with a non-diagnostic radiology-performed US for abdominal pain, who then required ORC for subsequent abdominal CT. We excluded children with a chronic gastrointestinal condition, a prior history of appendicitis, a history of allergy to ORC, a patient who had received ORC prior to arrival to the PED, and/or patients who had unstable vital signs.

### Methods

After we obtained informed written consent, patients received an emergency physician-performed POCUS evaluation, specifically imaging the abdominal aorta and RLQ structures (psoas muscle and iliac vessels) pre-ORC administration. Physicians obtained images, as per protocol, which entailed taking representative images in longitudinal and transverse orientation at the level of the cecum looking at the iliac vessels and psoas muscle and of the abdominal aorta. This was to ensure that differences in image quality would not be due to differences in technique or location. These images were recorded and stored.

**Figure 1:**
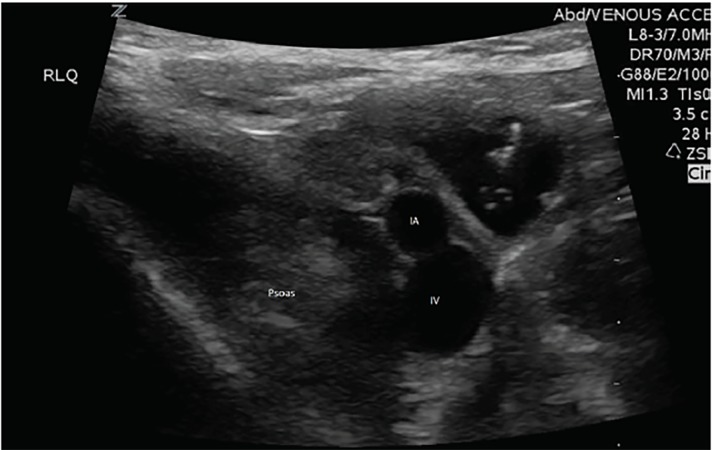
Pre-oral radio-contrast administration: psoas muscle and iliac artery (IA) and iliac vein (IV) labeled.

**Figure 2:**
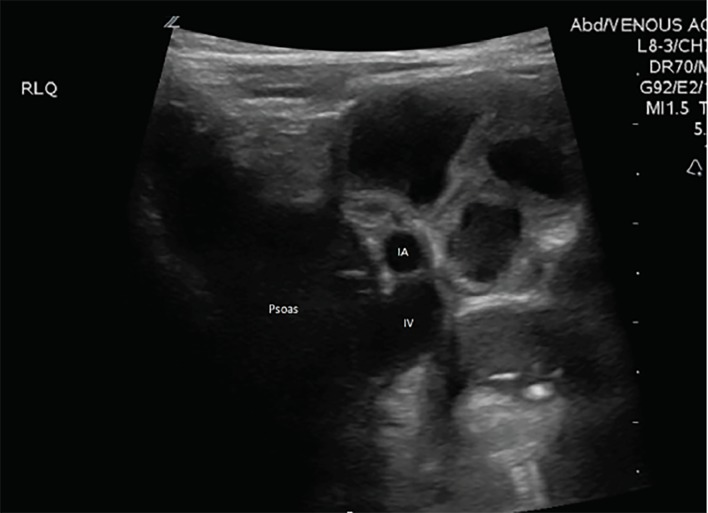
Post-oral radio-contrast administration: psoas muscle and iliac artery (IA) and iliac vein (IV) labeled.

Patients were then transported to the radiology department and received a formal radiology-performed abdominal US exam. Once the radiologist interpreted their study as non-diagnostic for appendicitis or other abdominal pathology, and the treating team felt the need to continue the diagnostic work up with a CT, ORC was ordered. The patient then received a weight-based dose of ORC, either diatrizoate meglumine 66%-diatrizoate sodium 10% and organically bound iodine (Gastroview) 366 milligrams organic iodine per milliliter (mgI/mL); OR iohexol 1.21 milligrams per milliliter (mg/mL) tromethamine and 0.1 mg/mL edetate calcium disodium and organically bound iodine (Omnipaque) 240 mgI/mL mixed with a weight-based amount of water or apple juice as per protocol, at time zero minutes. A repeat ED abdominal POCUS was performed by the same study physician who performed the initial ED POCUS in the same exact method between 90–120 minutes post-ORC administration. Once these images were recorded the study was concluded for that patient (Figures 1 and 2). The radiology-performed ultrasound had no bearing on the study parameters and was not assessed by the study team.

Additional data collected (Table 1) included the child’s age, gender, weight, height, body mass index, type and volume of contrast received, time interval between ORC administration and performance of US exams, volume and time of contrast ordered and ingested, and whether or not the patient vomited after drinking contrast.

Both pre- and post-ORC POCUS images were randomized with a non-descript code, and blinded physician-assessors were not aware which images were pre- or post-ORC. Individual subjects were not otherwise identifiable. The physician-assessors of the US images were fellowship-trained in point-of-care emergency ultrasonography and each had performed well over 1000 POCUS examinations, and over 10,000 quality assurance reviews of POCUS examinations. Assessors were blinded to all clinical details and identities and were not involved in recruitment of patients or image acquisition. All POCUS images were compared, evaluated and rated using a five-point Likert scale: 1 = not interpretable; 2 = barely interpretable; 3 = adequate for interpretation but of poor quality; 4 = interpretable and of average quality; 5 = interpretable and of superior quality.^2^

It was the goal of the assessors to determine whether the structures in question – the psoas muscle, iliac vessels and abdominal aorta – were either visible or not visible in each image. The assessors responsible for the blinded image review of the pre- and post-ORC POCUS studies did not perform any of the study ultrasounds on the subject patients. Again, the “formal” radiology-performed studies were not reviewed as they had no bearing on the study question.

### Statistical Analysis

A sample size calculation indicated that studying a minimum of 15 subjects’ POCUS exams would provide 80% power to detect at least a one-point Likert score difference (our minimal clinically significant difference) between mean pre- and post-ORC administration scores. Assuming a standard deviation of 1.25 and an effect size of 0.80m, we achieved a power of 83% by enrolling 17 patients.

We used SAS (Statistical Analysis System v9. SAS Institute, Cary, North Carolina) package for analysis of all results. A paired T-test was used to compare the mean difference in image quality scoring between pre- and post-ORC administered groups. A p-value <0.05 was considered statistically significant.

## RESULTS

**Figure 3:**
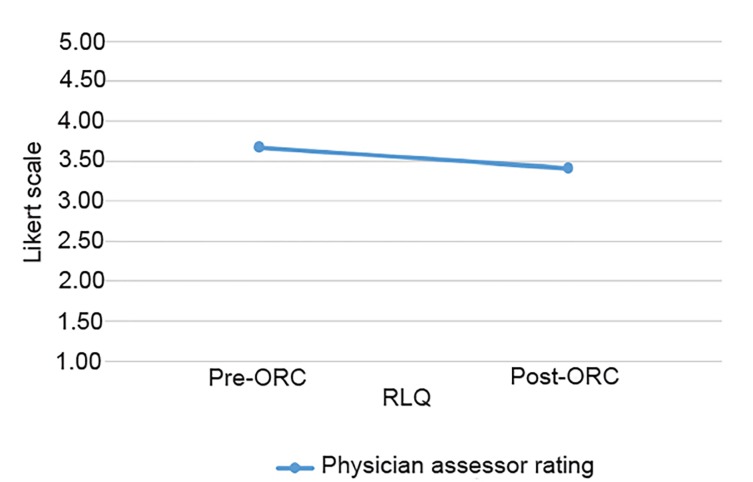
Mean physician-assessor scores of right lower quadrant (RLQ) ultrasound images. ORC, oral radio-contrast

**Figure 4:**
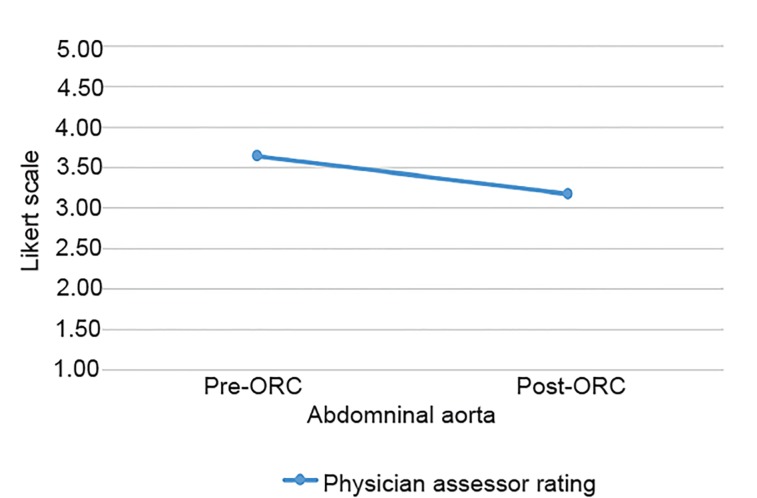
Mean physician-assessor scoring of abdominal aorta ultrasound images. ORC, oral radio-contrast.

Of the 17 patients enrolled in the study, all of them received two POCUS exams (pre- and post-ORC administration), each assessing the psoas muscle, iliac vessels and abdominal aorta. There was a total of 34 sonographic exams performed with two static images taken of the RLQ anatomy and the abdominal aorta, totaling 68 images for blinded review. The demographic profiles of study patients are given in Table 1. Figures 3 and 4 show no significant statistical differences noted for either RLQ structures or abdominal aorta image quality scoring.

Table 2 shows that although image quality was lessened after contrast, it was not significantly lessened. With regard to image quality, all post-ORC mean values were within 0.5 rating points of the pre-ORC mean values and, therefore, well within our predetermined range of a non-clinically significant difference (≤1 point on the Likert scale). Between the two physician assessors, there are four instances (24%) of differences of two on the Likert scale for aorta images compared to zero instances (0%) for the RLQ. Given these results, we can conclude that ORC does not significantly obscure abdominal US image quality.

## DISCUSSION

Patients presenting to the ED with abdominal pain often undergo diagnostic imaging, especially when attempting to determine whether appendicitis or other abdominal pathology is present. This often initially includes the performance of an abdominal US exam. When, as is frequently the case, the US result is non-diagnostic, performing advanced imaging (including abdominal CT) may be indicated. Optimally, abdominal CT is performed after ORC and maximized by waiting 90–120 minutes post-administration for contrast to transit to the lower reaches of the intestines.^3^,^4^ This can be time consuming, making it desirable to institute ORC as early as possible to maximize efficient patient flow and cycle time.^4^,^5^–^7^

Multiple imaging modalities can be used in diagnosing pediatric appendicitis, each with inherent risks and benefits. Historically, abdominal CT was favored due to its superior diagnostic accuracy. In children, sensitivity for the diagnosis of acute appendicitis by CT ranges between 94–100%^6^,^9^–^12^ with specificity at 93–100%. Subsequently, concern regarding the risk of CT ionizing radiation exposure and the potential for possibly developing a malignancy^13^,^14^ contributed to the increasing popularity of US.^15^ The benefits of US vs CT include lack of exposure to ionizing radiation, rapid performance, and relative inexpense.^15^ Additionally, US is often readily available throughout the day at many hospitals; and even more so as a point-of-care test that can be accurately performed by emergency physicians trained in this modality.^16^,^17^

When US is used but results are non-diagnostic, the next step in imaging is often the performance of abdominal CT. Children have a relatively lesser degree of intra-abdominal fat as compared to adults, which makes the distinction of periappendiceal fat-stranding relatively more difficult to detect on unenhanced CT. Thus, some experts recommend ORC prior to obtaining CT.^4^–^7^ Moreover, identification of other acute abdominal pathologic conditions may be enhanced using ORC.^3^ To achieve maximal quality images, it is recommended that CT be performed between 90–120 minutes after ORC administration to achieve optimal contrast delivery to the RLQ structures.

A commonly cited yet unsubstantiated clinical concern is the notion that ORC presence in the intestine can impair abdominal US image quality. This has prompted the practice of delaying the administration of ORC until after US completion. The only prior study related to this issue that we identified was by Dang et al,^18^ who recently reported results in adults who received ORC with comparison of abdominal US image quality pre- and post-ORC. They found no statistical or clinical difference in image quality obtained at each of the three time points: pre-ORC, followed by both one and two hours post-ORC.^18^ We know of no other study that similarly assesses this issue in children.

Similar to the results of the Dang study, our data likewise demonstrates no statistically or clinically significant differences in RLQ US image quality when obtained pre- and post-ORC administration. However, there is a possibility that aorta scans are affected clinically, but not statistically by ORC. We hypothesize that this could be due to the distention of bowel and gas caused by oral contrast creating a greater distance between the probe and the area of interest. It is important to note that aorta US is not widely done or used in the pediatric population when compared to the adult population and was included in this study solely for predictable anatomical location and location below the bowel. Thus, we feel ED protocols for diagnostically managing children with abdominal pain can allow for “early” administration of ORC, which can overlap with the clinical time necessary to obtain and interpret a radiology-performed US exam. Doing so could help minimize ED length of stay and allow for the expedited time to diagnosis. The implications of maximizing efficiency in patient flow includes improved metrics in ED throughput, superior patient satisfaction, and overall decrease in cycle time without compromising diagnostic accuracy in a busy ED setting.

## LIMITATIONS

This study was performed in a busy, single-center, diverse urban community with excellent integration of POCUS in the PED, which may limit its reproducibility to other centers. Many institutions either perform abdominal CT without the use of ORC or use other diagnostic modalities such as abdominal MRI to determine the case of abdominal pathology, making our study non-generalizable for these centers. Finally, we performed the study on a convenience sample limited by the number of recruiters that could be trained and the time of day that recruiters were present.

## CONCLUSION

Orally administered radiocontrast prior to performing an abdominal ultrasound in children with abdominal pain does not adversely affect US image quality. The early provision of ORC in children who may eventually require performance of an abdominal CT can maximize patient flow, cycle time, and ultimately diagnostic efficiency in an already busy pediatric ED setting.

## Figures and Tables

**Table 1:** Patient characteristics.

	N=17	
Mean age, years	10.3	±3.8
Male gender	10	59%
Mean weight, kg	41	±17.1
Mean height, cm	140.3	±24.2
Mean BMI	19.4	±5.1
Omnipaque^™^	12	71%
Gastroview^™^	4	29%
Median contrast ordered, ml	18	(12.8, 21.5)
Median total volume ordered, ml	360	(300, 475)
Vomiting	0	0%
Median time to drink contrast, min	15	(10, 22.5)
Median time to post-ORC US, min	95	(90,112.5)

**Table 2:** Comparison of ultrasound image quality of the right lower quadrant (RLQ) structures and abdominal aorta pre- and post- oral radio-contrast (ORC) administration.

Study	Pre-ORC	Post-ORC	P-value
RLQ structures (N=17)	3.68±0.81	3.41±0.66	0.132
Abdominal aorta (N=17)	3.65±0.81	3.18±0.90	0.060

Mean values (±standard deviation).

Results based on a 5-point Likert Scale (Norman G, 2010).
